# COPB1 deficiency triggers osteoporosis with elevated iron stores by inducing osteoblast ferroptosis

**DOI:** 10.1016/j.jot.2025.01.017

**Published:** 2025-03-21

**Authors:** Yike Wang, Ruizhi Zhang, Aifei Wang, Xiao Wang, Xiongyi Wang, Jiajun Zhang, Gongwen Liu, Kai Huang, Baoshan Liu, Yutong Hu, Sheng Pan, Xieyidai Ruze, Qiaocheng Zhai, Youjia Xu

**Affiliations:** a Department of Orthopaedics, The Second Affiliated Hospital of Soochow University, Suzhou, Jiangsu, China; b Department of Orthopaedics, Yancheng First Hospital, Affiliated Hospital of Nanjing University Medical School, Yancheng, Jiangsu, China; c Department of Orthopaedics, Suzhou TCM Hospital Affiliated to Nanjing University of Chinese Medicine, Suzhou, Jiangsu, China; d Orthopaedic Institute, Wuxi 9th People's Hospital Affiliated to Soochow University, Wuxi, Jiangsu, China; e Division of Spine Surgery, The Quzhou Affiliated Hospital of Wenzhou Medical University, Quzhou People's Hospital, Quzhou, China

**Keywords:** Osteoblast, Ferroptosis, Er stress, Proteomics, Targeted therapy

## Abstract

**Background:**

Osteoporosis (OP) is a systemic bone metabolic disease that results from an imbalance between bone formation and bone resorption. The accumulation of iron has been identified as an independent risk factor for osteoporosis. Ferroptosis, a novel form of programmed cell death, is driven by iron-dependent lipid peroxidation. Nevertheless, the precise role of ferroptosis in iron accumulation-induced osteoporosis remains uncertain.

**Methods:**

We utilized proteomics and ELISA to screen key regulatory molecules related to iron accumulation in osteoporosis populations. HE staining was used to assess osteocyte changes in Hamp knockout (KO) iron accumulation mouse models. Western Blot, qPCR, ALP staining, and Alizarin Red staining were employed to explore the effects of siRNA-mediated gene knockdown on osteogenic differentiation in the MC3T3 cell line. ELISA, micro-CT, von Kossa staining, toluidine blue staining, TRAP staining, and calcein analysis were used to study the bone phenotype of conditional gene knockout mice. RNA-seq, endoplasmic reticulum activity probes, transmission electron microscopy (TEM), Western Blot, co-immunoprecipitation (Co-IP), flow cytometry, and ChIP-seq were employed to investigate the regulatory mechanisms of the target gene in osteogenic differentiation. OVX and Hamp KO mice were used to establish osteoporosis models, and AAV-mediated overexpression was employed to explore the intervention effects of the target gene on osteoporosis.

**Results:**

The experiments demonstrate that iron accumulation can lead to changes in COPB1 expression levels in bone tissue. Cellular and animal experiments revealed that COPB1 deficiency reduces the osteogenic ability of osteoblasts. Transcriptome analysis and phenotypic experiments revealed that COPB1 deficiency induces ferroptosis and endoplasmic reticulum stress in cells. Further investigation confirmed that COPB1 plays a key role in endoplasmic reticulum stress by inhibits SLC7A11 transcription via ATF6. This reduces cystine uptake, ultimately inducing ferroptosis. Overexpression of COPB1 can restore osteogenic function in both cells and mice.

**Conclusion:**

This study elucidated the essential role of COPB1 in maintaining bone homeostasis and highlights it as a potential therapeutic target for treating iron accumulation-related osteoporosis.

**The translational potential of this article:**

Our data elucidate the critical role of COPB1 in maintaining bone homeostasis and demonstrate that COPB1 can directly promote bone formation, making it a potential therapeutic target for the future treatment of osteoporosis.

## Highlights

•Through human proteomics, COPB1 has been identified as a potential key target for osteoporosis caused by iron accumulation.
• COPB1 in osteoblasts has a fundamental role in bone homeostasis and its deletion induces osteopenia.
• COPB1-ATF6-SLC7A11 is a key pathway linking endoplasmic reticulum stress and ferroptosis.
• Overexpression of COPB1 can prevent osteopenia caused by iron accumulation or ovariectomy.

## Graphical abstract

**Figure:**
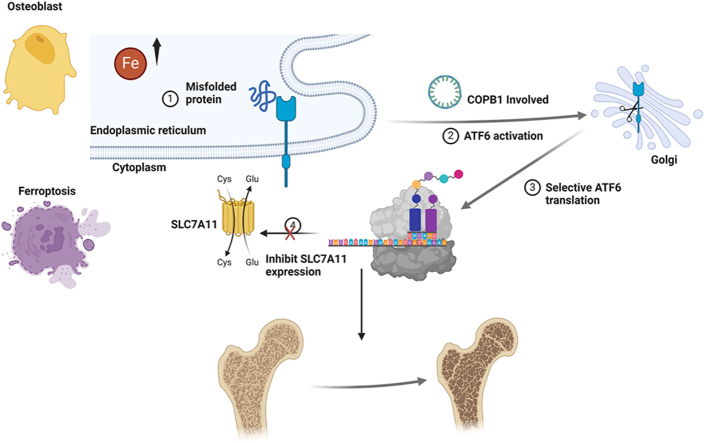

## 1 Introduction

Osteoporosis is a systemic bone metabolic disease marked by reduced bone density and degradation of bone microarchitecture, leading to increased fragility and fracture risk [1]. The primary pathological mechanism of osteoporosis involves is an imbalance in bone remodeling," where bone resorption surpasses bone formation [2]. Increasing evidence indicates that iron accumulation is an independent risk factor for osteoporosis [3,4]. Iron, as an essential element, participates in various fundamental processes at physiological concentrations, including regulating the proliferation and activation of osteoblasts and osteoclasts in bone tissue [5]. However, excessive iron, particularly ferrous ions, promotes the production of reactive oxygen species through the Fenton reaction. This process leads to oxidative stress and cytotoxicity, ultimately causing damage and death to relevant cells [6]. Although iron metabolism and bone metabolism are closely linked, the specific molecular mechanisms underlying iron accumulation-induced osteoporosis remain unclear.

Osteoblasts are target cells for iron action. A series of experiments have found that iron can inhibit the function and differentiation of osteoblasts through multiple pathways, including the transferrin receptor, Hedgehog, and FOXO pathways [[7], [8], [9]]. However, current research on iron accumulation and osteoporosis primarily focuses on cellular and mouse models, lacking clinical support and high-throughput mass spectrometry studies on human bone tissue samples. Proteins are the direct executors of biological functions, and proteomics, as a bioinformatics tool, can provide valuable information on biological functions and processes [10]. Studying proteomics in bone tissue is crucial because it is the direct site of action for osteoporosis.

Our team collected ten human bone tissue samples and conducted proteomics sequencing analysis. The analysis revealed significant changes in COPB1. COPB1, a vesicle transport protein, is primarily involved in transporting proteins between the Golgi apparatus and the endoplasmic reticulum. Studies have shown that COPI depletion in cancer cells leads to reduced cell survival rates and increased endoplasmic reticulum stress [11]. Previous studies have also shown that mutations in the gene encoding COPB1 cause severe developmental delays and microcephaly [12]. The COPI family member COPB2 plays a crucial role in regulating bone homeostasis by affecting the extracellular matrix of osteoblasts [13]. Despite these findings, the specific roles of the COPB family in bone remain unclear.

Ferroptosis is a unique form of programmed cell death driven by iron-dependent lipid peroxidation and is distinct from other forms of programmed cell death. Its key features include iron accumulation, lipid peroxidation, and the downregulation of glutathione peroxidase 4 [14]. The endoplasmic reticulum plays a crucial role in the correct folding, secretion, and processing of proteins. When unfolded proteins accumulate, they trigger the unfolded protein response, leading to endoplasmic reticulum stress [15]. If the stress is too intense or prolonged, endoplasmic reticulum cannot restore normal function, resulting in reduced cysteine uptake and increased intracellular iron accumulation. This imbalance affects the oxidative stress state of cells and ultimately causes ferroptosis [16]. Many studies have indicated that the abnormal death of osteoblasts is related to the occurrence and development of orthopedic diseases, including osteoporosis, osteoarthritis, and osteosarcoma [17,18]. However, the specific mechanisms remain unclear. Recent research suggests that the absence of COPB1 family members leads to endoplasmic reticulum stress [13]. Therefore, we hypothesize that COPB1 may regulate osteoblast function and differentiation through the ferroptosis mechanism under conditions of iron accumulation.

In this study, we found human bone tissue proteomics that COPB1 is a significant risk factor for osteoporosis with elevated iron stores. Cellular and mouse experiments revealed that COPB1 deficiency impairs osteogenesis. Mechanistically, reduced COPB1 expression induces ferroptosis through ATF6 regulation of SLC7A11. Moreover, targeting COPB1 significantly rescued osteogenic potential and prevented bone loss. Thus, we elucidate that COPB1 may be a critical pathogenic factor in osteoporosis with elevated iron stores, and intervening in COPB1 could be a potential therapeutic approach for treating this condition.

## 2 Materials and methods

### 2.1 Vivo experiments

#### 2.1.1 Human bone tissue sample preparation

The volunteers work was approved by the Second Affiliated Hospital of Soochow University under JD-LK-2020-027-0. All volunteers signed informed consent forms. All experiments described were performed in accordance with the ethical guidelines of the World Medical Association (Declaration of Helsinki). All participants were recruited from the Department of Orthopedics at The Second Affiliated Hospital of Soochow University in Suzhou City, Jiangsu Province, China. The inclusion criteria were patients with unilateral femoral neck fractures treated with hip replacement surgery, who underwent examination prior to total hip replacement. The exclusion criteria included infection, tumors, hip dysplasia, femoral head necrosis, osteochondrosis, a history of hip surgery, a history of anti-osteoporosis treatment, and other conditions affecting osteoporosis (e.g., thyroid disorders, parathyroid disorders, adrenal disorders, and pituitary disorders). A total of 10 individuals were included in the proteomics study, while 25 individuals were ultimately included in the serological testing. Patients were categorized into two groups based on their hip T-values and serum ferritin levels: the normal group (Group N) with T > −1.0 and Ferritin <200 nmol/mL, and the osteoporosis with elevated iron stores group (Group IOP) with T < −2.5 and Ferritin >200 nmoL/ml Table 1, Table 2.

**Table 1:** Comparison of baseline data between the normal bone mass group and the osteoporosis with elevated iron stores ($\bar{X}\pm S$).

	n	age (year)	BMI (kg/m^2^)	T-scores	
				hip	lumbar
N	5	79.2 ± 9.28	23.71 ± 4.39	0.06 ± 1.30	−1.22 ± 1.16
IOP	5	81.2 ± 7.73	19.34 ± 3.78	−3.64 ± 0.38 3	−3.48 ± 1.08
*P*	—	0.1233	0.0536	<0.001	<0.001

N: normal bone mass group, OP: osteoporosis with elevated iron stores, BMI: body mass index. *P* < 0.05 was considered statistically significant.

**Table 2:** Comparison of baseline data between the normal bone mass group and the osteoporosis with elevated iron stores ($\bar{X}\pm S$).

	n	age (year)	BMI (kg/m^2^)	Ferritin (ng/ml)	T-scores	
					hip	lumbar
N	15	66.3 ± 5.58	27.5 ± 3.74	90.09 ± 29.400	−0.06 ± 0.37	0.45 ± 0.77
IOP	10	68.2 ± 2.44	22.6 ± 2.70	277.40 ± 63.479	−2.99 ± 0.42	−3.27 ± 0.67
*P*	—	0.332	<0.001	<0.001	<0.001	<0.001

N: normal bone mass group, OP: osteoporosis with elevated iron stores, BMI: body mass index. *P* < 0.05 was considered statistically significant.

During surgery, an appropriate amount of femoral tissue was obtained from 0.5 cm below the cartilage. The samples were stored at −80 °C. Approximately 100 mg of tissue was removed using a trephine for protein mass spectrometry analysis.

#### 2.1.2 Animals

The study utilized COPB1 CKO mice, Hamp-KO mice, and female mice, all of which were provided by Jiangsu GemPharmatech Co. Ltd. (Jiangsu, China). All animal experiments were reviewed and approved by the Ethics Committee of Soochow University (approval number SUDA20200424A04). All animals were housed in an SPF-grade facility at Soochow University, where water and feed were freely available. Mouse tissues were collected at 8 and 24 weeks, respectively. The purchase and use of experimental animals were conducted in accordance with the guidelines for animal care at Soochow University, adhering to the standards of experimental animal management.

#### 2.1.3 Mouse model

By crossing homozygous mice carrying the OSX-Cre transgene with homozygous mice carrying the COPB1-Flox allele, we generated heterozygous offspring with or without the OSX-Cre allele. These offspring were further crossed to produce wild-type mice (Copb1), mice heterozygous for the OSX-Cre allele, and homozygous Copb1-flox mice with Copb1 deletion in the osteoblast lineage (Copb1flox/flox OSX-Cre). Genotypes were verified by PCR analysis of tail DNA samples. Tibial proteins were extracted to verify knockdown efficiency using Western blot. Hepcidin knockout mice were generated by mating mice carrying the Floxed Hamp allele, resulting in hepcidin knockout mice. Since hepcidin is the only hormone regulating iron metabolism in the body, hepcidin knockout mice serve as a model for studying endogenous iron accumulation [7]. Genotypes were verified by PCR analysis of tail DNA samples. For the ovariectomy (OVX) model, 8-week-old C57BL/6 female mice underwent bilateral ovariectomy.

#### 2.1.4 Immunohistochemistry staining

After sacrificing the mice with CO2, femoral tissues were collected and fixed in 4 % paraformaldehyde (PFA) for 48 h. The femurs were then decalcified in EDTA decalcification solution (Sloarbio, China) for 30 days. Subsequently, the decalcified samples underwent dehydration, clearing, and paraffin embedding. The decalcified samples were sectioned into 5-micron thick slices using a microtome. The sections underwent heat-mediated antigen retrieval for 20 min. After blocking with 5 % bovine serum for 30 min, the sections were incubated with the primary anti-COPB1 antibody (1:500) at 4 °C overnight. Subsequently, the sections were incubated with goat anti-rabbit secondary antibody (1:2000) at room temperature for 30 min. Immunostaining scores were determined by combining the intensity and area of staining.

#### 2.1.5 ELISA analysis

Serum from mice and human participants was collected. Mouse serum was obtained by collecting venous blood from the retro-orbital venous plexus after anesthetizing the mice with sodium pentobarbital. The collected blood was then centrifuged at 3000 rpm for 10 min at 4 °C, and the supernatant was collected for further use. The levels of β-CTX (Enzyme-linked Biotechnology, China), P1NP (Enzyme-linked Biotechnology, China), and COPB1 (Abbexa, USA) in the serum were measured using ELISA kits according to the manufacturer's instructions.

#### 2.1.6 Micro-CT analysis

Femurs from mice were fixed in 4 % PFA for 48 h and then analyzed using micro-CT. Femurs were scanned and analyzed for cortical and trabecular bone by micro-CT (SkyScan 1174, Bruker, Belgium). The acquisition parameters were as follows: X-ray voltage = 50 kV, X-ray current = 800 μA, filter = 0.5 mm aluminum, rotation step = 0.7°, and image pixel size = 10.3 μm. After scanning, images were reconstructed using NRecon software (Bruker, Belgium). Morphological analysis of trabecular bone was conducted by measuring bone mineral density (BMD), bone volume/tissue volume (BV/TV), trabecular thickness (Tb.Th), trabecular separation (Tb.Sp), trabecular number (Tb.N), and cortical thickness (Ct.Th). Three-dimensional reconstructions and bone renderings were generated using Mimics 14.0 imaging software(Materialise, Belgium).

#### 2.1.7 Bone histomorphometry

The femoral paraffin sections were stained with hematoxylin and eosin (HE) to analyze the number of osteocytes. Tartrate-resistant acid phosphatase (TRAP) staining was used to measure the number of osteoclasts in the femoral paraffin sections. Non-decalcified femoral tissue specimens were sectioned and stained with von Kossa silver solution (Solarbio, China). Non-decalcified femoral sections were also stained using a toluidine blue staining kit (Solarbio, China). One day and seven days before sacrificing the mice, calcein (20 mg/kg) was administered via intraperitoneal injection. Subsequently, non-decalcified femoral sections were prepared, and fluorescence signals were collected using a fluorescence microscope. Histomorphometric analysis was performed using ImageJ software (NIH, USA).

#### 2.1.8 Biomechanical testing

Mouse tibias were stored at −80 °C and subsequently subjected to three-point mechanical testing using a universal testing machine (CellScale, Canada). Maximum load (N), stiffness (N/mm), and failure energy (J) were calculated from the displacement–load curves.

#### 2.1.9 Mouse AAV9 overexpression virus injection

Copb1 overexpression virus (pAAV-CMV-Copb1) were prepared by OBiO Technology (Shanghai, China). A single dose of the virus (1.07 E+13) was injected via the tail vein into male hepcidin knockout mice (8 weeks old) and OVX mice (8 weeks old). Eight weeks after the procedure, the mice were sacrificed. Western blot was performed using tibia and liver tissues to assess the efficiency of COPB1 overexpression.

#### 2.1.10 Prussian Blue staining

Liver paraffin sections were stained to detect iron content using the Prussian Blue Iron Stain Kit (Solarbio, China) according to the manufacturer's instructions.

### 2.2 Vitro experiments

#### 2.2.1 Cell culture

The MC3T3-E1 cell line was purchased from Procell (Hubei, China). MC3T3-E1 cells are a pre-osteoblastic cell line derived from the calvaria of mice, commonly used to study the molecular mechanisms of osteoblast differentiation and bone formation. MC3T3-E1 cells were cultured in α-MEM medium (Procell, China) supplemented with 10 % FBS (Procell, China) and 1 % PS(Procell, China). The cells were maintained in a cell incubator at 37 °C with 5 % CO2, and the medium was changed every other day. FAC (Sigma, USA) was used to simulate an iron accumulation environment in vitro.

#### 2.2.2 RNA extraction and qPCR

Total RNA was extracted from cells or bone tissues using TRIZOL (Invitrogen, USA). For bone samples, the tissue was ground into a powder in liquid nitrogen. The powdered bone or cells were collected in an Eppendorf tube, and TRIZOL was added. After lysing at room temperature for 3 min, RNA was extracted according to the manufacturer's instructions.

Reverse transcription of RNA was performed using the PrimeScript RT reagent kit. cDNA (1 μg) was used for qPCR with SYBR Premix Ex Taq (Takara, Japan). The sequences of the qPCR primers are listed in the table Table 3.

**Table 3:** Primers used for quantitative RT-PCR.

Gene	Primers(Forward/Reverse)
*Actin*	5′-CATTGCTGACAGGATGCAGAAGG-3′
	5′-TGCTGGAAGGTGGACAGTGAGG-3′
*Runx2*	5′-CCTGAACTCTGCACCAAGTCCT-3′
	5′-TCATCTGGCTCAGATAGGAGGG-3′
*Alp*	5′-CCAGAAAGACACCTTGACTGTGG-3′
	5′-TCTTGTCCGTGTCGCTCACCAT-3′
Ocn	5′-GCAATAAGGTAGTGAACAGACTCC-3′
	5′-CCATAGATGCGTTTGTAGGCGG-3′
*Osterix*	5′-GGCTTTTCTGCGGCAAGAGGTT-3′
	5′-CGCTGATGTTTGCTCAAGTGGTC-3′
*Copb1*	5′-TCTTGTCCGTGTCGCTCACCAT-3′
	5′-TCTTGTCCGTGTCGCTCACCAT-3′
*Ptgs2*	5′-GCGACATACTCAAGCAGGAGCA-3′
	5′-AGTGGTAACCGCTCAGGTGTTG-3′
*Atf6*	5′-GTCCAAAGCGAAGAGCTGTCTG-3′
	5′-AGAGATGCCTCCTCTGATTGGC-3′
*Lc3b*	5′-GTCCTGGACAAGACCAAGTTCC-3′
	5′-CCATTCACCAGGAGGAAGAAGG-3′
*Gsdmd2*	5′-GGTGCTTGACTCTGGAGAACTG-3′
	5′-GCTGCTTTGACAGCACCGTTGT-3′
*Casp8*	5′-ATGGCTACGGTGAAGAACTGCG-3′
	5′-TAGTTCACGCCAGTCAGGATGC-3′

#### 2.2.3 Western Blot

Bone tissue powder ground in liquid nitrogen or collected cells were lysed in RIPA lysis buffer containing protease inhibitors (Roche, USA) and phosphatase inhibitors (Roche, USA) on ice for 30 min. After centrifugation, the supernatant was collected, mixed with loading buffer, and denatured at 95 °C for 5 min. The denatured protein samples were subjected to SDS-PAGE and then transferred to a Polyvinylidene Fluoride Membrane. The membrane was blocked with 5 % non-fat milk at room temperature for 1 h and incubated with primary antibodies at 4 °C overnight. The primary antibodies used at a 1:1000 dilution were as follows: ALPL (Affinity, China), RUNX2 (Affinity, China), OSTERIX (Huabio, China), OCN (Affinity, China), BMP2 (Abcam, UK), SMAD5 (CST, USA), P-SMAD1/5 (CST, USA), WNT10b (Affinity, China), β-CATENIN (Huabio, China), ATF6, IRE1 (CST, USA), P-IRE1 (CST, USA), P-PERK (CST, USA), GPX4 (Abcam, UK), SLC7A11 (Huabio, China), P62 (CST, USA), LC3A/B (CST, USA), Cleaved-CASPASE3 (CST, USA), BECLIN1 (CST, USA), HSP90 (Proteintech, China), GAPDH (Proteintech, China), ACTIN (Huabio, China). The membrane was then incubated with anti-rabbit IgG or anti-mouse IgG secondary antibodies at 1:5000 at room temperature for 1 h. Protein expression was detected using a chemiluminescence imaging system. Each experiment used at least three independently prepared protein extracts. Band intensity was quantified using ImageJ software (NIH,USA).

#### 2.2.4 siRNA knockdown

MC3T3-E1 cells were transfected using Hieff Trans siRNA (Yeasen, China) when they reached 80 % confluence. After 72 h of transfection, cellular RNA was extracted, and qPCR was used to assess gene knockdown efficiency. The sequences of the siRNA were as follows:

siRNA Copb1 forward: 5′-GCCACAACUCUAACCAAGAUUTT-3′, reverse: 5′-AAUCUUGGUUAGAGUUGUGGCTT-3′

siRNA Atf6 forward: 5′-GCUGUCUGUGUGAUGAUAGUA-3′, reverse: 5′-UACUAUCAUCACACAGACAGC-3′

#### 2.2.5 ALP and alizarin red staining

MC3T3-E1 cells were seeded in 6-well plates. When the cells reached 80 % confluence, the medium was changed to an osteogenic induction medium containing α-MEM supplemented with 50 μg/mL ascorbic acid, 10 mM β-glycerophosphate, 10 % FBS, and 1 % PS. The medium was replaced every two days. On the 7th and 14th days of induction, the cells were fixed with 4 % paraformaldehyde. Alkaline Phosphatase detection and 1 % Alizarin Red S staining were performed according to the manufacturer's instructions, and images were captured under a microscope.

#### 2.2.6 Cell proliferation assay

MC3T3-E1 cells were seeded in 96-well plates (100 cells per well). After 3 days of COPB1 knockdown treatment, each well was supplemented with a solution containing fresh medium (90 μl) and CCK-8 reagent (10 μl) (Dojindo, JAPAN). The cells were then incubated at 37 °C for 2 h in the dark, followed by the addition of stop buffer. Absorbance at 450 nm was measured using a microplate reader (Thermo, USA). All procedures were performed according to the manufacturer's instructions.

#### 2.2.7 Intracellular Fe^2+^ determination

According to the manufacturer's instructions, the cellular ferrous iron content was measured using the Cellular Ferrous Ion Assay Kit (Elabscience, China). After knocking down ***Copb1*** for 3 days, the cells were washed with PBS and subsequently lysed using a lysis buffer. The cell lysate was collected, followed by the addition of the chromogenic solution, and incubated at 37 °C for 10 min. Finally, the absorbance of each sample was measured at 593 nm using a microplate reader, and the iron concentration in the samples was calculated based on the standard curve of the iron concentration gradient.

#### 2.2.8 Endoplasmic reticulum fluorescence detection

Cells were seeded in chamber dishes three days in advance. When the cells reached 70 % confluence, they were washed three times with HBSS and stained with an endoplasmic reticulum staining working solution according to the manufacturer's instructions. Observations and photographs were taken using a confocal microscope (Olympus, JAPAN) with excitation at 488/565 nm.

#### 2.2.9 Transmission electron microscopy analysis of endoplasmic reticulum and mitochondrial morphology

MC3T3-E1 cell pellets were fixed in 2.5 % glutaraldehyde solution. The morphology of the endoplasmic reticulum and mitochondria was analyzed using transmission electron microscopy at the HaoKe Testing Center (Zhejiang, China) following standard procedures.

#### 2.2.10 Detection of malondialdehyde, reactive oxygen species, and lipid peroxides

Three days after Copb1 knockdown, cells were washed once with PBS and lysed on ice using RIPA lysis buffer for 30 min. The lysates were then centrifuged at 12,000 rpm for 10 min. The supernatants were collected and analyzed for malondialdehyde (MDA) content using an MDA assay kit (Dojindo, Japan) according to the manufacturer's instructions with a microplate reader. Reactive oxygen species (ROS) and lipid peroxides (LPO) were detected using ROS and LPO assay kits (Dojindo, Japan). Cells from the control group and the COPB1 knockdown group were seeded in 12-well plates. The next day, the supernatant was removed, and the cells were washed twice with HBSS. After washing, the cells were collected and incubated with ROS and LPO staining working solutions for 30 min. Detection was then performed using a flow cytometer (BD, USA) with an FITC filter.

#### 2.2.11 Cystine uptake assay

Cystine uptake was measured using a cystine uptake assay kit (Dojindo, Japan) according to the manufacturer's instructions. Cells from the control group and the COPB1 knockdown group were seeded in black 96-well plates. The next day, the medium was replaced with the specified medium, and after washing the cells with PBS, the working solution was added. After incubating for 30 min, fluorescence was measured using a fluorescence microplate reader (Biotek, USA). Cell suspensions without cysteine analogs were used as blanks. The excitation wavelength was set to 485 nm and the emission wavelength to 535 nm. The protein concentration of each treatment group was measured to normalize the corresponding fluorescence intensity.

#### 2.2.12 Immunoprecipitation

For immunoprecipitation, cells were lysed at 4 °C with lysis buffer containing 100× protease inhibitors and 100× phosphatase inhibitors. Protein G Sepharose Beads (GE, USA) were blocked overnight. The lysates were incubated overnight with anti-ATF6, anti-COPB1, or IgG antibodies. The next day, 20 μL of beads were added to the protein lysates and incubated at 4 °C for 2 h. The beads were then boiled and analyzed for COPB1 and ATF6 expression via Western Blot.

#### 2.2.13 Live and dead cell assay

Following 48-h treatment with Erastin or RSL3, live and dead cells were detected using the Calcein AM/PI Double Staining Kit (Elabscience, China) according to the manufacturer's instructions, with flow cytometry and fluorescence microscopy.

#### 2.2.14 Chromatin immunoprecipitation (ChIP)

Cells were fixed with 1 % formaldehyde at 4 °C for 10 min, then washed with PBS, and nuclei were extracted. The nuclei were lysed in 1 % Sodium Dodecyl Sulfate and sonicated to shear the chromatin DNA into fragments of 100–400 bp. After removing cell debris, 10 μL was taken as input. The supernatant was pre-incubated with magnetic beads containing IgG (Invitrogen, USA) for 2 h, followed by overnight incubation with magnetic beads containing anti-ATF6 antibody. The samples were then washed with low salt wash buffer, high salt wash buffer, LiCl wash buffer, and TE buffer. After reversing the crosslinks, the DNA was purified using the MinElute PCR Purification Kit (Qiagen, Germany).

#### 2.2.15 Establishment of stable cell lines

Copb1 overexpression lentivirus and control vector lentivirus Vector were prepared by OBiO Technology (Shanghai, China). Stable overexpression of Copb1 in MC3T3-E1 cells was achieved by transfecting the lentivirus followed by puromycin selection. The efficiency of lentiviral overexpression in cells was assessed using Western blot.

#### 2.2.16 Apoptosis detection

Three days after Copb1 knockdown, cells were collected and apoptosis was detected using the Annexin V-FITC/PI Apoptosis Kit (Elabscience, China) according to the manufacturer's instructions.

### 2.3 Proteomics, RNA-sequencing, and ChIP-sequencing

Proteomics data analysis of the 10 collected bone tissue samples was performed using SIMCA software (Umetrics, Sweden). RNA-seq analysis was conducted using the Dr. Tom System (https://biosys.bgi.com) by BGI Genomics Co, China. ChIP-seq analysis was carried out using MACS2 software (Department of Bioinformatics and Computational Biology, USA) to detect peaks and identify enriched IP regions with a default threshold of q value ≤ 0.05. The results were then analyzed to show the distribution of chromosome localization, peak width, enrichment level, significance level, and the number of peak summits relative to peak start positions.

### 2.4 Statistical analysis

Statistical analysis was conducted using GraphPad software (version 9.01). Statistical methods included t-tests or one-way analysis of variance (ANOVA). A P value < 0.05 was considered statistically significant. All data are presented as mean ± standard error of the mean (SEM). All representative experiments were repeated at least three times.

## 3 Results

### 3.1 Correlation between COPB1 and osteoporosis with elevated iron stores

**Fig. 1:**
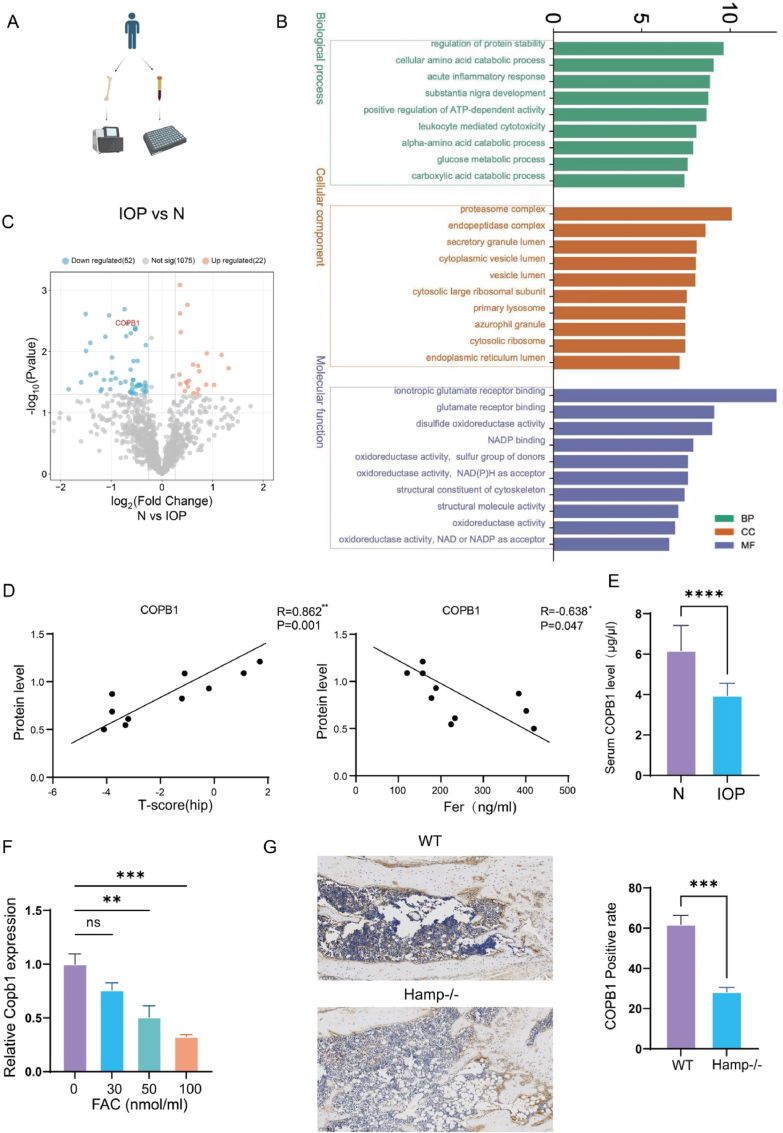
Correlation between Copb1 and iron accumulation in osteoporosis. a. Schematic diagram representing bone tissue and serum in the osteoporosis with elevated iron stores group and the normal iron and bone mass group. b. GO pathway enrichment analysis of differentially expressed proteins between the IOP and P groups (n = 5). c. Volcano plot showing differentially expressed proteins between the IOP group and the N group (n = 5). d. Correlation analysis of hip joint T-scores, serum ferritin, and COPB1 protein levels (Pearson correlation analysis) (n = 10). e. Elisa analysis of serum COPB1 levels between the IOP group (n = 10) and the N group (n = 15). f. qPCR analysis of COPB1 compared to the control group (n = 3). g. Representative images and quantification of femoral COPB1 IHC staining in Hamp-ko mice and WT mice (n = 3) (scale bar, bottom right).ns = not significant, ∗P < 0.05, ∗∗P < 0.01, ∗∗∗P < 0.001, ∗∗∗∗P < 0.0001.

To explore the relationship between iron accumulation and osteoporosis, we collected serum and bone tissue samples from 10 postmenopausal women who had undergone hip replacement surgery due to hip fractures(Fig. 1A). We divided these patients into two groups: five with osteoporosis with elevated iron stores (IOP) and five with normal bone mass (N). Detailed clinical information for these patients is provided in Table 1. We extracted proteins from the bone tissues and conducted a proteomic analysis. This analysis identified 2900 proteins, of which 1150 were quantifiable and exceeded a specific expression threshold (Figs. S1A and B). Differential expression analysis revealed 22 upregulated proteins and 53 downregulated proteins (Fig. 1C). Among these 75 differentially expressed proteins, 66 were identified in osteoblast cell lines, as indicated by the UniProt database. Gene Ontology (GO) enrichment analysis demonstrated that these proteins were significantly involved in functions related to protein stability and were predominantly located in the endoplasmic reticulum lumen and ribosomal subunits(Fig. 1B).

Previous research suggests that double mutations in COPB1, which is involved in ER-Golgi transport, can lead to microcephaly. Based on these findings, we directed our subsequent research towards the COPB1 protein. Our analysis revealed that COPB1 expression in bone tissue is significantly and negatively correlated with both bone density and the severity of iron accumulation in patients(Fig. 1D). To exclude potential biases caused by serum ferritin measurement, we further assessed serum iron levels. The results similarly demonstrated that the expression level of COPB1 in bone tissue was significantly and negatively correlated with bone density and serum iron concentration in patients (Fig. S1C). Additionally, we collected serum samples from both the IOP and N groups. ELISA results demonstrated that serum levels of COPB1 were markedly lower in the IOP group compared to the N group (Fig. 1E).

To investigate whether COPB1 plays a role in the osteoblast response to iron accumulation, we established a cellular iron accumulation model using the MC3T3-E1 cell line. The results demonstrated a significant decrease in Copb1 mRNA levels correlated with increased cellular iron content (Fig. 1F). Similarly, knockdown of ***Copb1*** resulted in a significant decrease in intracellular ferrous iron content (Fig. S1D). Hepcidin, the only known hormone that negatively regulates iron metabolism, is absent in Hepcidin knockout mice (Hamp-ko/Hamp−/−), which serve as an endogenous model for iron accumulation with reduced bone mass(Figs. S1E and F). Immunohistochemistry of femurs from Hamp-ko mice showed decreased expression of COPB1(Fig. 1G).

In summary, these findings suggest that COPB1 responds to iron accumulation, and its expression level is associated with osteoporosis with elevated iron stores.

### 3.2 COPB1 deficiency inhibits osteoblastogenesis in vitro

**Fig. 2:**
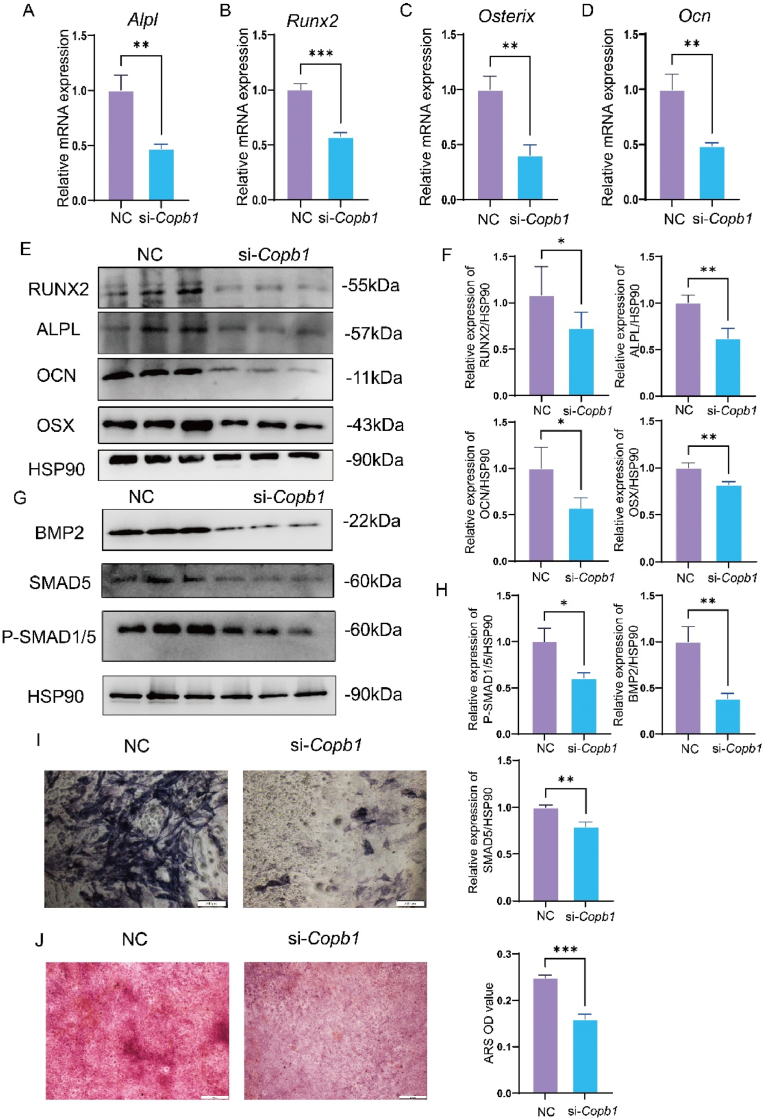
Copb1 deficiency inhibits in vitro osteogenesis. a-d. qPCR analysis of osteogenesis-related genes (Alpl, Runx2, Osterix, Ocn) in MC3T3-E1 cells in the control group and knockdown group after 7 days of osteogenic differentiation induction. e-f. Western Blot analysis and quantitative statistical analysis of osteogenesis-related proteins (ALPL, RUNX2, OSTERIX, OCN) in MC3T3-E1 cells in the control group and knockdown group after 7 days of osteogenic differentiation induction. g-h. Western Blot analysis and quantitative statistical analysis of osteogenesis-related pathway proteins (BMP2, SMAD5, P-SMAD1/5) in MC3T3-E1 cells in the control group and knockdown group after 7 days of osteogenic differentiation induction. i. Representative images of alkaline phosphatase staining of MC3T3-E1 cells in the control group and knockdown group after 7 days of osteogenic differentiation induction (scale bar 200 μm). j. Representative images of alizarin red staining of MC3T3-E1 cells in the control group and knockdown group after 14 days of osteogenic differentiation induction, with quantitative analysis performed using a microplate reader after dissolving the stain (scale bar 200 μm). n = 3. Compared to the control group, ∗P < 0.05, ∗∗P < 0.01, ∗∗∗P < 0.001.

Previous research identified Copb1 as a potential regulatory factor in osteoporosis with elevated iron stores. To assess its role in osteogenesis, we used siRNA to knock down Copb1 in the MC3T3-E1 cell line (Fig. S2A). On the seventh day of osteogenic induction, we measured the expression of osteogenesis-related genes by qPCR. Compared to the control group, Copb1 deficiency led to a downregulation of osteogenic markers, including Alpl, Runx2, Osterix, and Osteocalcin(Fig. 2A–D). Western blot analysis confirmed that the protein levels of these osteogenic markers corresponded to the observed changes in RNA levels(Fig. 2E and F). Furthermore, ALP and Alizarin Red staining indicated that Copb1 deficiency significantly reduced ALP activity and mineralization in MC3T3-E1 cells (Fig. 2I and J).

The BMP signaling pathway is known to be crucial for regulating osteogenic differentiation. We observed that COPB1 deficiency attenuated the expression of proteins involved in the BMP2-SMAD pathway (Fig. 2G and H).

These results suggest that COPB1 knockdown inhibits osteogenic differentiation and mineralization in MC3T3-E1 cells, likely by downregulating the BMP2-SMAD signaling pathway.

### 3.3 COPB1 Deletion in Osteoblasts Leads to bone loss

**Fig. 3:**
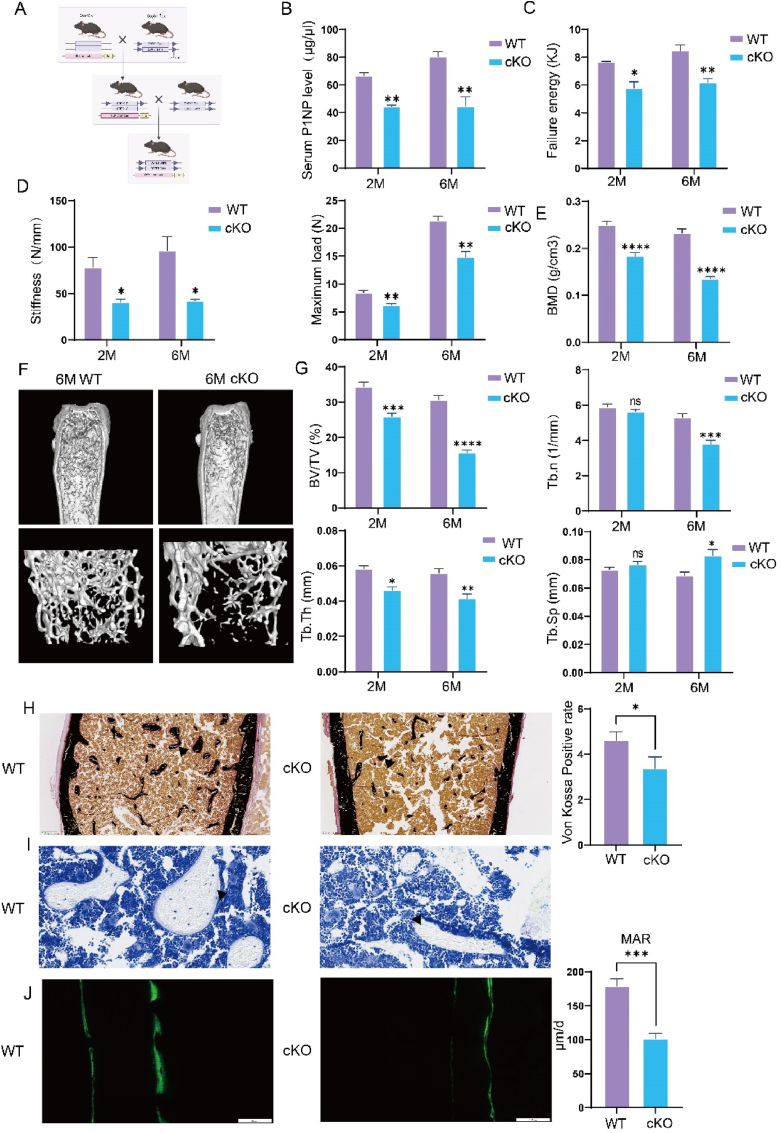
COPB1 deletion in osteoblasts leads to bone loss. a. Schematic diagram of COPB1 CKO mouse breeding. b. Serum levels of type I collagen amino-terminal propeptide (P1NP) in 2-month-old and 6-month-old COPB1 CKO and WT male mice (n = 3). c-d. Quantitative analysis of maximal load, fracture energy, and stiffness in the three-point bending test of tibia bone midshafts from 2-month-old and 6-month-old male mice (n = 3). e, g. Histomorphometric analysis of trabecular bone in 2-month-old and 6-month-old COPB1 CKO and WT male mice, including bone mineral density (BMD), bone volume per tissue volume (BV/TV), trabecular thickness (Tb.Th), trabecular number (Tb.N), and trabecular separation (Tb.Sp) (n = 6). f. Representative micro-CT images of whole femurs (bottom) and trabecular bone (bottom) from 6-month-old COPB1 CKO and WT male mice. h. Representative images and quantitative analysis of Von Kossa staining in 6-month-old COPB1 CKO and WT male mice (n = 3) (scale bar in the bottom left). i. Representative images of toluidine blue staining in 6-month-old COPB1 CKO and WT male mice (n = 3) (scale bar in the bottom left). j. Representative images and quantitative analysis of calcein double labeling in 6-month-old COPB1 CKO and WT male mice (n = 3) (scale bar 100 μm).Compared to the control group, ns = not significant, ∗P < 0.05, ∗∗P < 0.01, ∗∗∗P < 0.001, ∗∗∗∗P < 0.0001.

To further investigate the role of COPB1 in osteoblasts, we generated an osteoblast-specific COPB1 knockout mouse model (Copb1flox/flox OSX-Cre, referred to as CKO) (Fig. 3A). Western blot analysis revealed that Copb1 expression in the tibia was almost completely absent(Fig. S2D). Additionally, we noted that the deletion of Copb1 specifically in osteoblasts did not affect the fertility of the mice and had no impact on their physique(Fig. S2C).

We assessed the levels of the bone formation marker Procollagen Type I N-terminal Propeptide (P1NP) and found that serum P1NP levels were significantly lower in 2-month-old COPB1 CKO mice, with the difference becoming more pronounced by 6 months(Fig. 3B). In contrast, the serum levels of the bone resorption marker C-terminal telopeptide of type I collagen (β-CTX) were similar between COPB1 CKO and wild-type (WT) mice at both 2 and 6 months(Fig. S2B). This finding suggests that bone resorption activity remained unchanged.

We next evaluated the biomechanical properties of bones using a three-point bending test. The results revealed that the maximum load, stiffness, and energy to failure of the tibiae in COPB1 CKO mice were significantly reduced at both 2 and 6 months of age(Fig. 2C and D). Micro-CT analysis showed decreased trabecular bone mineral density (BMD) and bone volume fraction (BV/TV) in 2-month-old COPB1 CKO mice, along with a reduced trabecular number (Tb.n). By 6 months, this trend of bone loss continued, with changes in trabecular thickness (Tb.th) and trabecular separation (Tb.sp) reflecting reduced bone mass(Fig. 2E–G).

Von Kossa staining demonstrated a marked decrease in mineralization levels(Fig. 2H). Morphological analysis further revealed a significant reduction in both the number and surface area of osteocytes in COPB1 CKO mice (Fig. 2I) (Fig. 2J). Additionally, calcein double labeling indicated a significant reduction in the mineral apposition rate of trabecular bone in COPB1 CKO mice, suggesting impaired bone formation(Fig. 2J). Tartrate-resistant acid phosphatase (TRAP) staining showed no significant difference in osteoclast numbers between COPB1 CKO and control mice(Fig. S2E).

These findings suggest that the bone loss observed in osteoblast-specific COPB1 knockout mice is likely due to impaired osteoblast-mediated bone formation.

### 3.4 COPB1 Deficiency Induces Endoplasmic Reticulum Stress and Ferroptosis

**Fig. 4:**
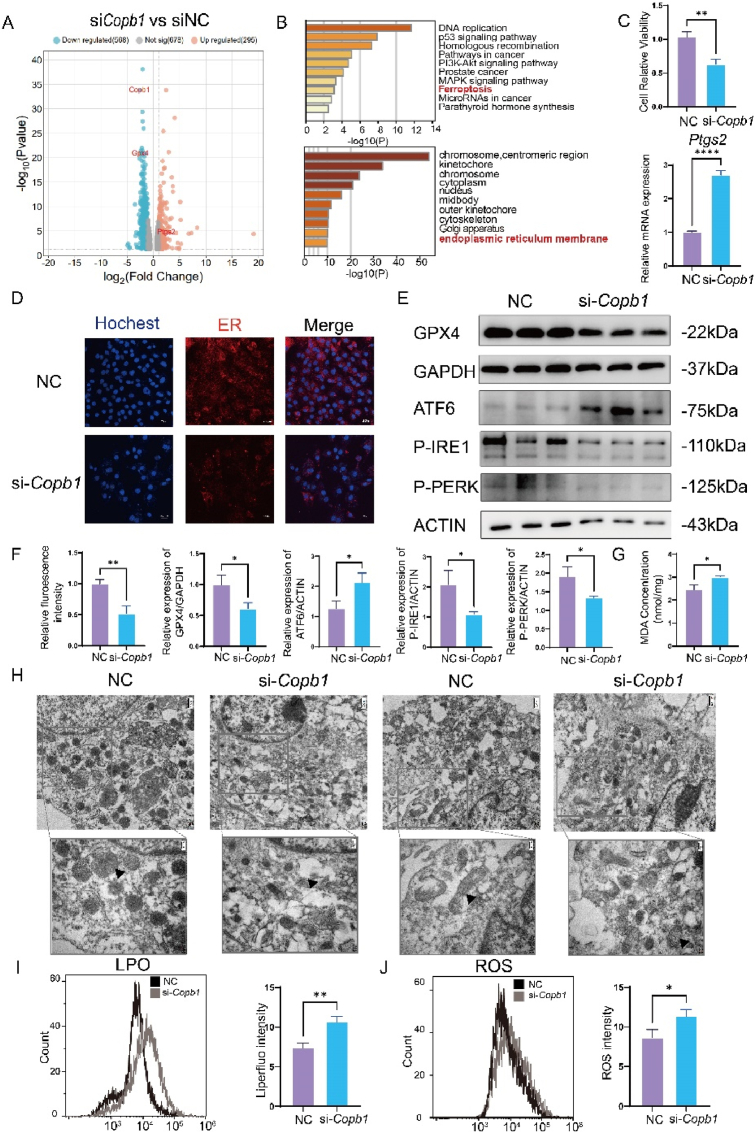
COPB1 deficiency induces endoplasmic reticulum stress and ferroptosis. a. Volcano plot showing differentially expressed genes in MC3T3-E1 cells between the control group and the COPB1 knockdown group(n = 3). b. KEGG pathway enrichment analysis of differentially expressed proteins between the NC and si-COPB1 groups (n = 3). c. qPCR analysis of the ferroptosis-related gene (Ptgs2) and CCK-8 assay for live cell count after 3 days of Copb1 knockdown in MC3T3-E1 cells (n = 3). d, f. Endoplasmic reticulum fluorescence intensity detected and quantitatively analyzed using an endoplasmic reticulum fluorescent probe in MC3T3-E1 cells after 3 days of Copb1 knockdown (n = 3, blue indicates nucleus, red indicates endoplasmic reticulum). e-f. Western Blot analysis and quantitative statistical analysis of GPX4, ATF6, P-IRE1, and P-PERK expression levels in MC3T3-E1 cells in the control and knockdown groups (n = 3). g. Microplate reader detection of MDA content in MC3T3-E1 cells in the control and knockdown groups (n = 3). h. Transmission electron microscopy observation of endoplasmic reticulum and mitochondrial morphology in MC3T3-E1 cells after 3 days of Copb1 knockdown (n = 3) (scale bar 200 nm, 500 nm). i. Flow cytometry detection and quantitative analysis of LPO content in MC3T3-E1 cells in the control and knockdown groups (n = 3). j. Flow cytometry detection and quantitative analysis of ROS content in MC3T3-E1 cells in the control and knockdown groups (n = 3). Compared to the control group, ∗P < 0.05, ∗∗P < 0.01, ∗∗∗∗P < 0.0001.

To explore how COPB1 regulates osteogenesis, we knocked down COPB1 in MC3T3-E1 cells using siRNA and performed RNA-seq analysis. Our results showed 908 genes were downregulated (>2-fold) and 634 genes were upregulated (>2-fold) (Fig. 4A). Kyoto Encyclopedia of Genes and Genomes(KEGG) pathway enrichment analysis of these differentially expressed genes indicated significant enrichment in ferroptosis-related signaling pathways(Fig. 4B). GO analysis revealed enrichment of these genes in components of the endoplasmic reticulum (ER) membrane(Fig. 4B). Since COPB1 is involved in vesicle transport between the ER and the Golgi apparatus, we hypothesized that COPB1 deficiency might alter ER morphology. Using an ER probe and confocal microscopy, we observed a significant decrease in ER fluorescence intensity in COPB1-deficient cells, suggesting disrupted ER structure(Fig. 4D).

To confirm that COPB1 knockdown induces ferroptosis, we examined the expression of key ferroptosis-related mRNA and proteins using qPCR and Western blotting. Our results showed elevated Ptgs2 mRNA levels and significantly decreased GPX4 protein levels(Fig. 4C E and F). We also observed notable cell death in COPB1-deficient cells(Fig. 4C).

Previous studies have shown that mitochondrial morphology changes are hallmarks of ferroptosis and that COPB1 deficiency affects ER function. Using transmission electron microscopy, we observed abnormal expansion and swelling of the ER in COPB1-deficient cells, with the disappearance of normal folded structures and a significant reduction in ribosomes on the rough ER(Fig. 4H). The mitochondria displayed membrane shrinkage and transitioned from “long rod-like” to “dot-like” shapes, which are typical characteristics of ferroptotic mitochondria(Fig. 4H) [19]. Additionally, Western blot analysis revealed significantly increased expression of the ER stress key protein ATF6, while the expression of other critical ER stress pathway proteins, P-IRE1 and P-PERK, decreased(Fig. 4E and F).

Malondialdehyde (MDA), lipid peroxides (LPO), and ROS are crucial compounds involved in ferroptosis [20]. We measured MDA using an enzyme-linked immunosorbent assay (ELISA) and assessed LPO and ROS levels using flow cytometry. Our results showed significant increases in MDA, LPO, and ROS levels in COPB1-deficient cells(Fig. 4G I and J). When we added a ferroptosis inhibitor (Fer-1) to COPB1-deficient cells during osteogenic differentiation, the expression of osteogenic differentiation-related proteins increased(Fig. S3D). This suggests that COPB1 inhibits osteogenic differentiation through the ferroptosis pathway.

To rule out the possibility that COPB1 knockdown affects osteoblasts through other cell death mechanisms, we examined the expression of autophagy-related proteins (P62, LC3A/B, BECLIN1) and the apoptosis key protein cleaved-CASPASE3. We also assessed apoptotic cells using flow cytometry. These markers showed no changes, indicating that autophagy and apoptosis were not involved(Figs. S3A–C).

In summary, our results demonstrate that COPB1 deficiency induces ER stress and ferroptosis, ultimately impairing osteogenic differentiation.

### 3.5 COPB1 induces ferroptosis through endoplasmic reticulum stress

**Fig. 5:**
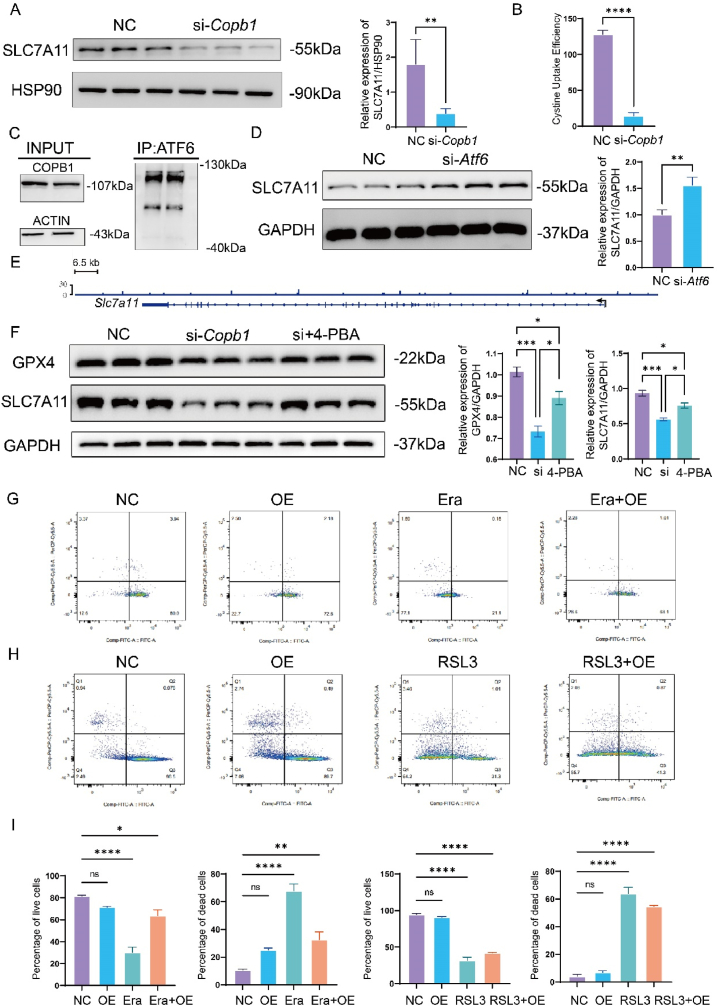
COPB1 induces ferroptosis through endoplasmic reticulum stress. a. Western Blot analysis and quantitative statistical analysis of SLC7A11 expression levels in MC3T3-E1 cells in the control and knockdown groups (n = 3). b. Microplate reader detection of cystine transport efficiency in MC3T3-E1 cells in the control and knockdown groups (n = 3). c. CO-IP results of COPB1 using ATF6 antibody. d. Western Blot analysis and quantitative statistical analysis of SLC7A11 expression levels in MC3T3-E1 cells after ATF6 knockdown (n = 3). e. CHIP-seq peaks of ATF6 enrichment in the DNA region of SLC7A11. f. Western Blot analysis and quantitative statistical analysis of SLC7A11 and GPX4 expression levels in MC3T3-E1 cells in the control, knockdown, and rescue groups (n = 3). g, i. Flow cytometry detection and quantitative analysis of live and dead cell numbers in MC3T3-E1 cells after overexpression of COPB1 and addition of Erastin (n = 3). (NC represents the control group, OE represents the COPB1 overexpression group, Era represents the addition of Erastin, and Era + OE represents the group with both addition of Erastin and COPB1 overexpression.)h, i. Flow cytometry detection and quantitative analysis of live and dead cell numbers in MC3T3-E1 cells after overexpression of COPB1 and addition of RSL3 (n = 3). (NC represents the control group, OE represents the COPB1 overexpression group, RSL3 represents the addition of RSL3, RSL3+OE represents the group with both addition of RSL3 and COPB1 overexpression.).

Our previous research suggested that COPB1 deficiency might lead to ER stress and ferroptosis. We found that the absence of COPB1 reduced the expression of the cystine transporter protein SLC7A11(Fig. 5A). Using an enzyme-linked assay, we confirmed that the cellular cystine uptake capacity decreased(Fig. 5B). Under normal conditions, ATF6 resides in the ER in an inactive form and is transported to the Golgi apparatus for processing during ER stress. Co-immunoprecipitation experiments demonstrated that COPB1, a key protein in ER-Golgi transport, interacts with ATF6, suggesting a relationship between these proteins(Fig. 5C).

Previous studies have shown that ER stress can lead to ferroptosis. When we knocked out Atf6 in cells, we observed a significant increase in SLC7A11 protein expression(Fig. 5D S4A and B). Given that activated ATF6 translocates to the nucleus to regulate the transcription of various ER stress-related proteins, we performed CHIP-seq to investigate its role as a transcription factor. GO pathway enrichment analysis indicated changes in cellular transport functions(Fig. S4C). Using IGV to analyze the results, we found binding peaks of ATF6 in the intronic regions of SLC7A11, suggesting that ATF6 may inhibit SLC7A11 expression as a transcription factor(Fig. 5E).

4-Phenylbutyric acid (4-PBA) is known as a selective inhibitor of ER stress. We added 4-PBA to cells experiencing ER stress induced by COPB1 knockdown. Our results showed that inhibiting ER stress effectively restored the expression of GPX4 and SLC7A11 proteins(Fig. 5F). We then used recognized ferroptosis inducers, RSL3 (which directly reduces GPX4 expression) and Erastin (which inhibits SLC7A11 transport), in subsequent experiments. We established a stable COPB1 overexpression cell line and treated it with RSL3 and Erastin(Fig. S4D). Flow cytometry and immunofluorescence analyses revealed that COPB1 overexpression significantly mitigated Erastin-induced ferroptosis but had a less pronounced effect on RSL3-induced ferroptosis(Fig. 5G–I S4E and F).

These findings suggest that the COPB1-ATF6-SLC7A11 pathway is critical in mediating ER stress-induced ferroptosis through iron accumulation.

### 3.6 Overexpression of COPB1 rescued bone mass in osteoporosis with elevated iron stores

**Fig. 6:**
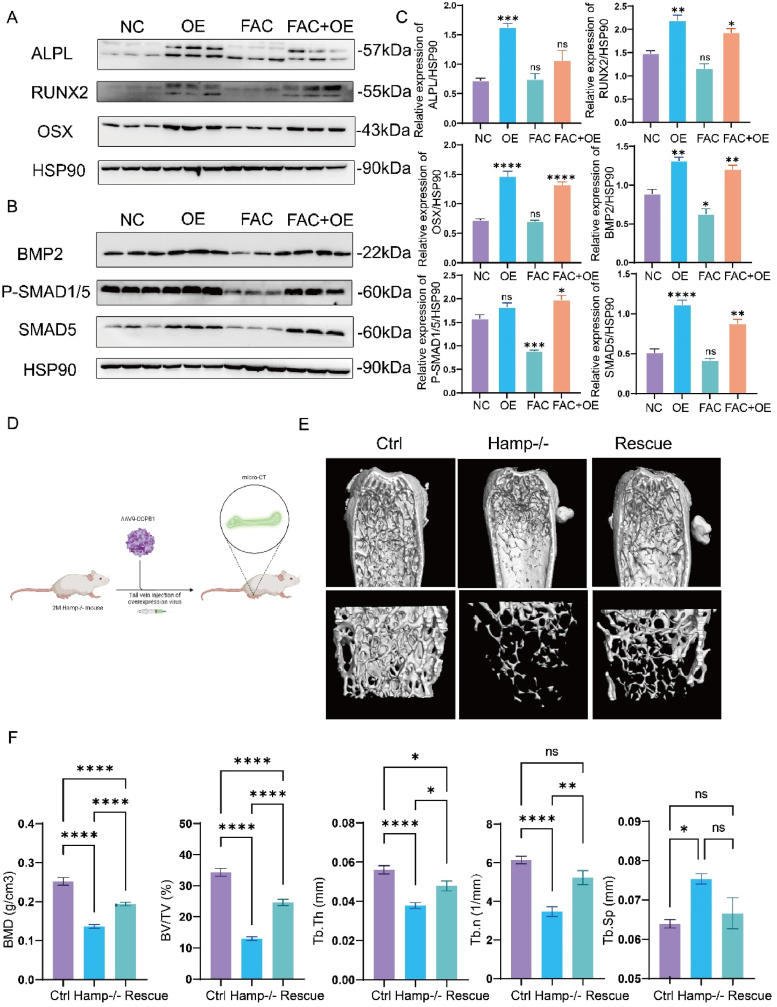
Overexpression of COPB1 restores bone loss induced by iron accumulation. a, c. Western Blot analysis and quantitative statistical analysis of ALPL, RUNX2, and OSX expression levels in MC3T3-E1 cells in the control, COPB1 overexpression, iron accumulation, and groups (n = 3). (NC represents the control group, OE represents the COPB1 overexpression group, FAC represents the intracellular iron accumulation group, and FAC + OE represents the group with both intracellular iron accumulation and COPB1 overexpression.)b, c. Western Blot analysis and quantitative statistical analysis of BMP2, P-SMAD1/5, and SMAD5 expression levels in MC3T3-E1 cells in the control, overexpression, iron accumulation, and rescue groups (n = 3). (NC represents the control group, OE represents the COPB1 overexpression group, FAC represents the intracellular iron accumulation group, and FAC + OE represents the group with both intracellular iron accumulation and COPB1 overexpression.)d. Schematic diagram of the rescue experiment in Hamp−/− mice. e. Representative micro-CT images of whole femurs (bottom) and trabecular bone (bottom) from 4-month-old Ctrl, Hamp−/−, and Rescue male mice(Rescue represents COPB1 overexpression.). f. Histomorphometric analysis of trabecular bone in 4-month-old Ctrl, Hamp−/−, and Rescue male mice, including bone mineral density (BMD), bone volume per tissue volume (BV/TV), trabecular thickness (Tb.Th), trabecular number (Tb.N), and trabecular separation (Tb.Sp) (n = 6) (Rescue represents COPB1 overexpression.).

Our previous research identified COPB1 as a potential key regulator in osteoporosis with elevated iron stores. To further investigate its potential for treating this condition, we first established a stable cell line overexpressing COPB1. Western blot and qPCR analyses revealed that COPB1 overexpression significantly restored the expression of osteogenic markers ALPL, RUNX2, and Osterix, which had been downregulated due to iron accumulation(Fig. 6A and C S5A). Additionally, COPB1 overexpression alone enhanced the expression of these osteogenic markers, consistent with earlier findings that COPB1 knockdown impairs osteogenesis. This overexpression also activated the BMP-SMAD signaling pathway (Fig. 6B and C).

Previous studies have demonstrated that adeno-associated virus serotype 9 (AAV9) has high transfection efficiency in osteogenic lineage cells, including osteoblasts and osteocytes (Fig. 6D). Therefore, we administered AAV9-COPB1 via tail vein injection in mice. Two months post-injection, we observed increased COPB1 protein levels in the tibia (Figs. S6B and C). Micro-CT analysis showed that AAV9-COPB1 intervention significantly rescued bone mass in Hamp-ko mice, as indicated by increases in BMD, BV/TV, Tb.Th, and Tb. n (Fig. 6E and F). We also developed an OVX osteoporosis model and found that tail vein injection of AAV9-COPB1 partially restored bone mass in OVX mice (Figs. S6D and E).

These results suggest that overexpression of COPB1 in bone tissue promotes osteogenic differentiation and helps alleviate osteoporosis caused by iron accumulation.

## 4 Discussion

In recent years, an increasing number of studies have identified iron accumulation as an independent risk factor for osteoporosis [3]. Our previous research found a significant negative correlation between bone density and both iron content in bone tissue and serum ferritin levels [21]. Other studies have similarly noted that in disuse osteoporosis induced by weightlessness, bone density correlates negatively with ferritin levels [22]. It is currently understood that iron accumulation primarily results from the lack of menstrual iron loss in postmenopausal women, leading to a reduction of approximately 36 mg of iron excretion per year and resulting in systemic iron accumulation [21]. As a systemic circulatory change, iron accumulation can induce osteoporosis by affecting various bone metabolism-related cells. However, current research on the relationship between iron accumulation and osteoporosis is mostly limited to cellular and animal models, and the discovery of key pathogenic factors mainly relies on non-clinical samples.

COPB1 is a key component of the COPI complex and is highly conserved across evolution, primarily playing a role in intracellular vesicular transport [11]. Previous studies have demonstrated that the COPI family is critical in bone formation. Mutations in COPB2, a member of the COPI family, have been associated with osteoporosis and fractures, with mechanisms linked to disruptions in collagen transport mediated by the endoplasmic reticulum and Golgi apparatus [13]. In our study, we conducted a proteomic analysis of bone tissue proteins from 10 human samples and identified COPB1 as a potential key factor in osteoporosis with elevated iron stores. Further research showed that the loss of Copb1 leads to an increase in intracellular iron levels. In both hepcidin-knockout iron-overloaded mouse models and osteoblasts treated with iron in vitro, excessive iron ions were found to suppress Copb1 expression. Additionally, studies have reported that the loss of COPZI can induce increased intracellular iron levels [18]. Existing literature indicates that the Golgi apparatus and endoplasmic reticulum work synergistically through vesicle transport to participate in the synthesis, processing, and transport of iron metabolism-related proteins, thereby maintaining intracellular iron homeostasis [23]. Any dysfunction in vesicle transport or these organelles could result in iron metabolism disorders. Therefore, we hypothesize that COPB1 may serve as a critical intermediary in the regulation of iron metabolism.

The core issue of osteoporosis is typically caused by an imbalance between bone resorption and bone formation, resulting in bone loss due to disrupted bone remodeling. Previous studies have suggested that blocking COPI-mediated vesicle transport can induce apoptosis of mature osteoclasts and impair their bone resorption function [24]. However, this finding is inconsistent with our proteomic and serological data. Therefore, we hypothesize that the relationship between the COPI family and osteoporosis is more likely related to bone formation involving osteoblasts. To validate this hypothesis, we conducted cell experiments and animal models, both of which demonstrated the critical role of COPB1 in bone formation. Considering that current treatments for osteoporosis mainly include bisphosphonates, selective estrogen receptor modulators (SERMs), and RANKL inhibitors, which primarily focus on inhibiting osteoclast activity, there are relatively few strategies directly aimed at restoring the osteogenic potential of osteoblasts [25]. We propose that overexpressing COPB1 could directly promote bone formation. Further cell experiments demonstrated that overexpressing COPB1 not only restored the impaired osteogenic capacity caused by iron overload but also significantly enhanced the osteogenic potential of cells. These findings suggest that COPB1 could be a novel therapeutic target for treating osteoporosis associated with elevated iron levels.

The adeno-associated virus (AAV) vectors offer unique advantages in preclinical and clinical studies due to their high transfection efficiency, ability to provide sustained gene expression, and low immunogenicity and pathogenicity [26]. In mice, tail vein injection of AAV virus enables targeted intervention in bone tissue [27]. Our study demonstrates that COPB1 delivered via AAV9 can restore bone mass loss in postmenopausal female mice and iron-accumulation mice. These findings also suggest that COPB1 plays a greater role in promoting bone formation by osteoblasts compared to its effect on osteoclast-related bone formation. Previous studies have shown that bone loss in OVX mice is caused by ferroptosis in bone cells [28]. This study demonstrates that the overexpression of COPB1 can reverse the ferroptosis phenotype in these cells. This may be a potential mechanism by which COPB1 overexpression restores bone mass in OVX mice. Previous research suggests that COPI complex deficiency could impact vesicular transport, leading to abnormal activation of the STING signaling pathway [29]. The STING signaling pathway plays a dual role in bone metabolism through different activation modes: activation of the STING/IFN-β signaling pathway reduces bone resorption by inhibiting osteoclast differentiation, while activation of the STING/NF-κB signaling pathway promotes osteoporosis by increasing bone resorption and decreasing bone formation [30,31]. Additionally, activation of STING inhibits the formation of H-type blood vessels, which possess osteogenic potential, thereby suppressing bone formation [32]. The STING inhibitor C-176 alleviates osteolytic diseases associated with osteoclasts by inhibiting osteoclast differentiation [33]. This could represent another potential mechanism through which COPB1 overexpression in mouse bone tissue restores bone mass.

Ferroptosis is a type of iron-dependent programmed cell death [14]. Studies show that ferroptosis can worsen osteoporosis by affecting various bone cells, including osteoblasts and osteoclasts [34,35]. Our preliminary research demonstrated that iron accumulation regulates Nox4 transcription via iron response elements [36]. COPI family genes can regulate ferroptosis through multiple pathways. In tumor cells, the knockdown of the COPI family member COPZ1 leads to an increase in nuclear receptor coactivator 4 (NCOA4), which subsequently elevates ferroptosis levels [37]. However, considering the role of COPI in mediating vesicle transport between the ER and the Golgi apparatus, current studies on the COPI family have primarily focused on its regulation of ferroptosis, with limited exploration of its role in ER functions. Furthermore, as ER stress can trigger ferroptosis, we aim to elucidate how COPB1 induces ER stress to promote ferroptosis through this study.

Through in vitro studies, we confirmed that COPB1 plays a crucial role in ER stress through vesicular transport. COPB1 deficiency led to a significant decrease in ER activity and structural damage to the ER. Previous research shows that the COPB1 complex is essential for maintaining material exchange between the Golgi apparatus and the ER and that ATF6 activation requires transport to the Golgi for cleavage into its active form [38]. Our CO-IP results reveal that COPB1 interacts with ATF6, suggesting that ATF6 processing and activation may directly involve COPB1.

Persistent ER stress may also cause negative feedback regulation of the PERK and IRE1 signaling pathways to prevent excessive suppression of protein synthesis [39]. Our Western blot analysis revealed a decrease in the expression of proteins involved in the IRE1 and PERK pathways, which aligns with the severe structural damage to the ER observed through confocal microscopy. We observed that SLC7A11 expression decreases under ER stress conditions and that knocking down ATF6 in cells also reduces SLC7A11 expression. Further CHIP-seq analysis showed that ATF6, as a transcription factor, binds to the intronic regions of SLC7A11 DNA. This finding suggests that ER stress can regulate the transcription of ferroptosis-related genes through the ATF6 pathway. Increasing evidence indicates that transcription factor enrichment in intronic regions may form transcriptional repressor complexes that physically block RNA polymerase extension, thereby reducing transcription efficiency [40,41]. Additionally, it has been reported that ATF6 can downregulate gene expression through transcriptional repression [42,43]. We further explored the use of the ER stress inhibitor 4-PBA and found that inhibiting ER stress partially restores ferroptosis induced by COPB1 deficiency. This finding is consistent with others, which demonstrated that inhibiting ER stress can suppress ferroptosis [44]. Flow cytometry analysis revealed that overexpression of COPB1 can restore ferroptosis caused by inhibited cysteine uptake, while it has little effect on ferroptosis induced solely by the suppression of antioxidant activity.

However, this study has several limitations. First, although we performed proteomic sequencing on human bone tissue samples, the sample size remains relatively small. Additionally, since most volunteers undergoing hip replacement surgery are elderly, our study lacks data from younger volunteers. Furthermore, all our volunteers were from the same hospital in a single region, which may limit the generalizability of the study's findings. Therefore, future studies should expand the sample size and include young volunteers from different regions and ethnic groups to validate our results. Currently, studies on osteoporosis associated with elevated iron stores primarily focus on postmenopausal women, while data on male volunteers are relatively scarce. To broaden the scope of research, future studies should include male volunteers with iron accumulation to investigate the relationship between bone mass and iron accumulation more comprehensively.

## Author contributions author-disclosure

Y.W., Qiaocheng Zhai and Youjia Xu designed the study. Youjia Xu and Xiao Wang provided funding support. Y.W. and R.Z. performed most of the experiments. A.W. and G.L. conducted bioinformatics analysis. X. W and B.L. collected clinical samples. J.Z. and Y.H. helped with genotyping and mouse samples collection. X.R. and S.P. performed micro-CT and helped with analyzing the data. Y.W. and Qiaocheng Zhai wrote the manuscript. K.H. and Youjia Xu reviewed and revised the manuscript. All authors read and approved the finial version of manuscript.

## Informed consent statement

Informed consent was obtained from all subjects involved in the study.

## Funding

This work was supported by the National Natural Science Foundation of China (82372455); Jiangsu Provincial Medical Key Laboratory Cultivation Unit (JSDW202254); Suzhou Revitalizing Healthcare with Science and Education Youth Science and Technology Project (KJXW2021050).

## Declaration of competing interest

The authors declare that have no competing interests.
