# Supplementary material for: COPB1 deficiency triggers osteoporosis with elevated iron stores by inducing osteoblast ferroptosis
Source: J Orthop Translat. 2025 Mar 21;51:312–28. doi: 10.1016/j.jot.2025.01.017 (PMC11981772; doi:10.1016/j.jot.2025.01.017)
Supplement: Multimedia component 1 [file mmc1.docx]

**
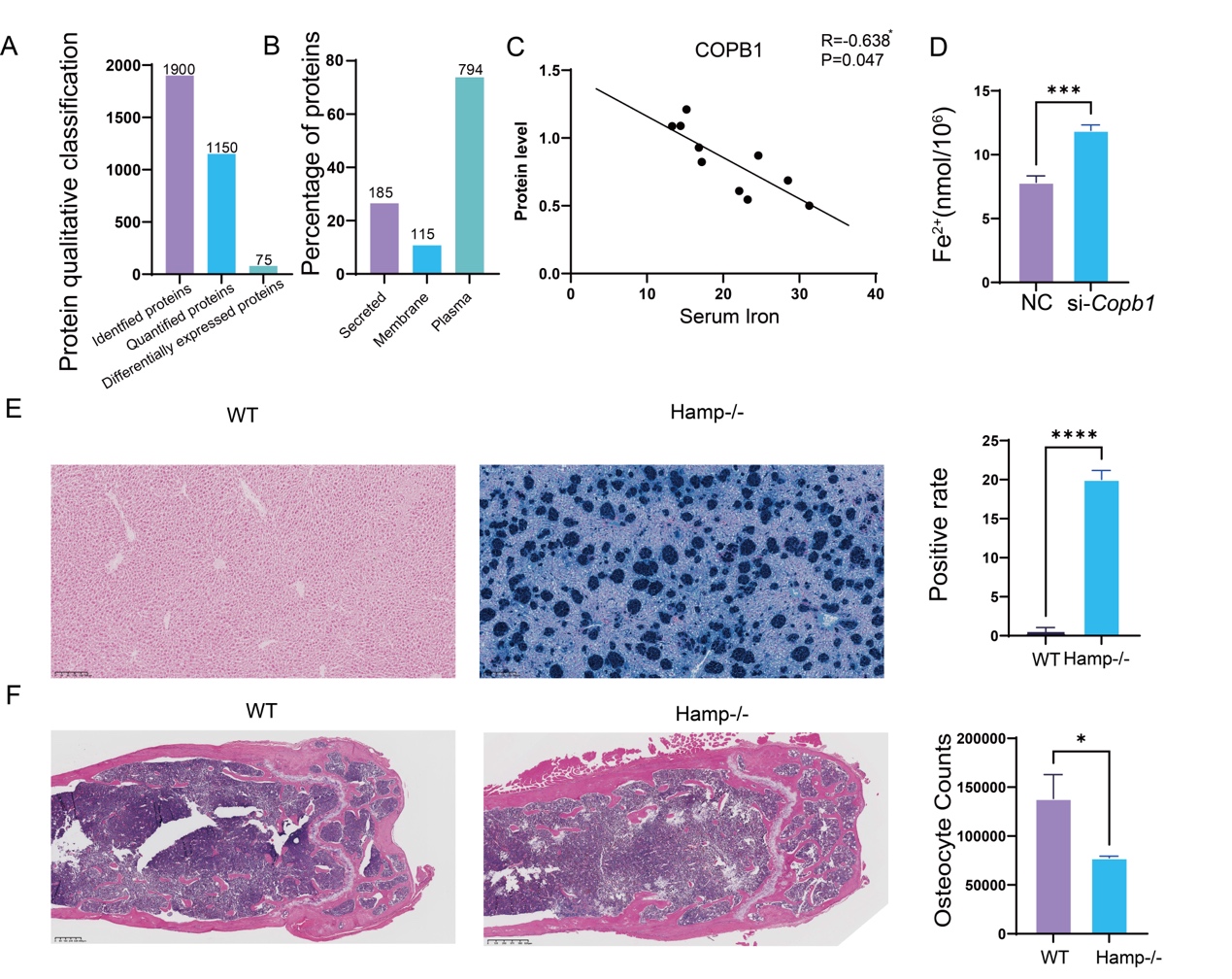
**

**Supplementary Figure 1**

a. Classification of total protein quantification in human bone tissue proteomics. b. Functional localization distribution of the identified proteins. c. Correlation analysis of serum iron, and COPB1 protein levels (Pearson correlation analysis) (n=10). d. The intracellular ferrous iron content after Copb1 knockdown three days in MC3T3-E1 cells (n=3).e. Prussian blue staining and quantitative statistical analysis of the liver from Hamp-/- and WT mice (n=3). f. HE staining of the distal femur from Hamp-/- and WT mice and quantitative statistical analysis of osteocyte numbers (n=3).


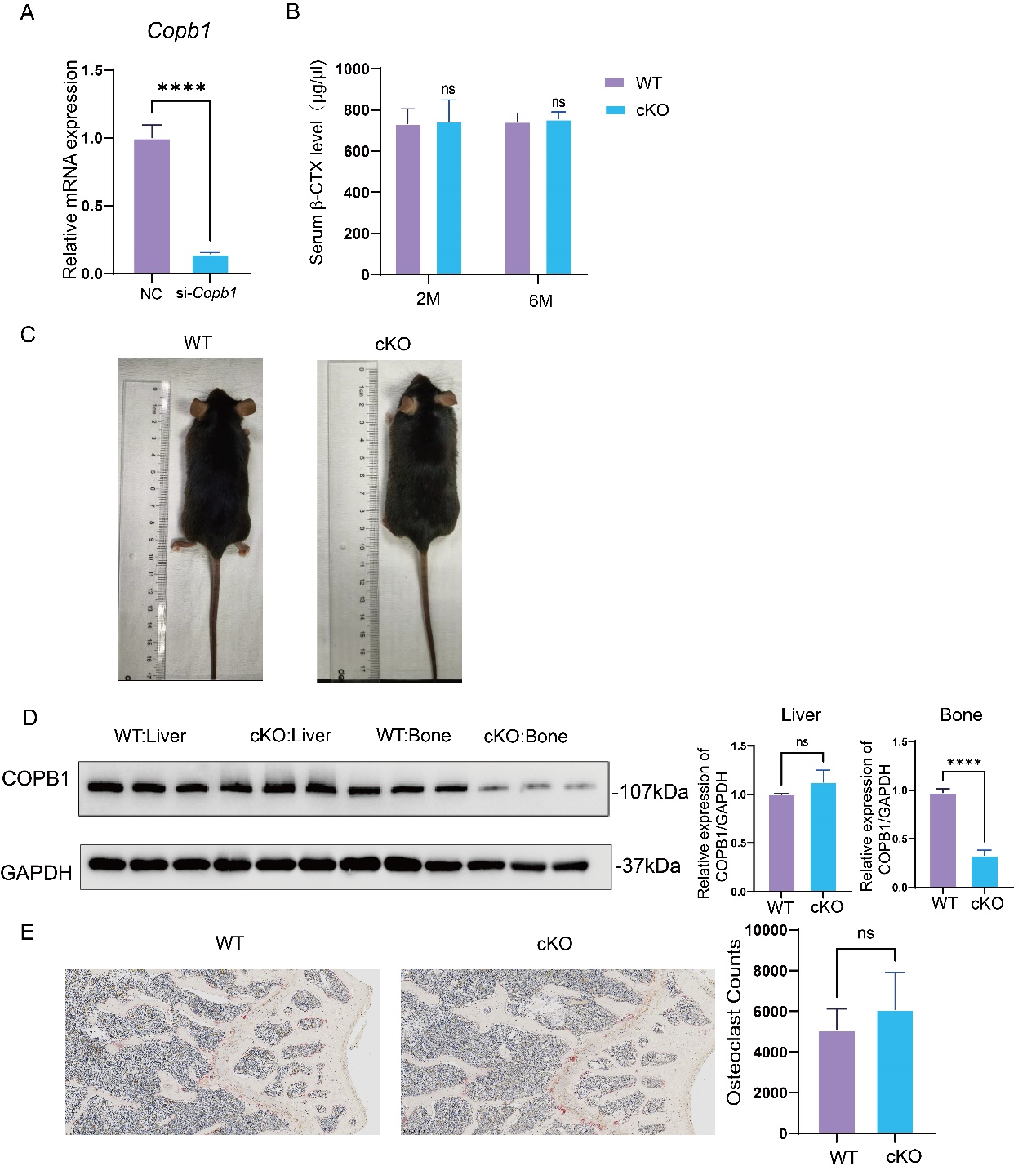


**Supplementary Figure 2**

a. qPCR analysis of knockdown efficiency after three days of siRNA-mediated *Copb1* knockdown in MC3T3-E1 cells (n=3). b. Serum levels of type I C-terminal telopeptide (β-CTX) in 2-month-old and 6-month-old COPB1 CKO and WT male mice (n=3). c. Comparison of body length between 6-month-old COPB1 CKO and WT male mice. d. Western Blot analysis and quantitative statistical analysis of COPB1 expression levels in MC3T3-E1 cells in the liver and bone tissue of WT and CKO mice (n=3). e. TRAP staining results and quantitative statistical analysis of the distal femur from 6-month-old COPB1 CKO and WT male mice (n=3).


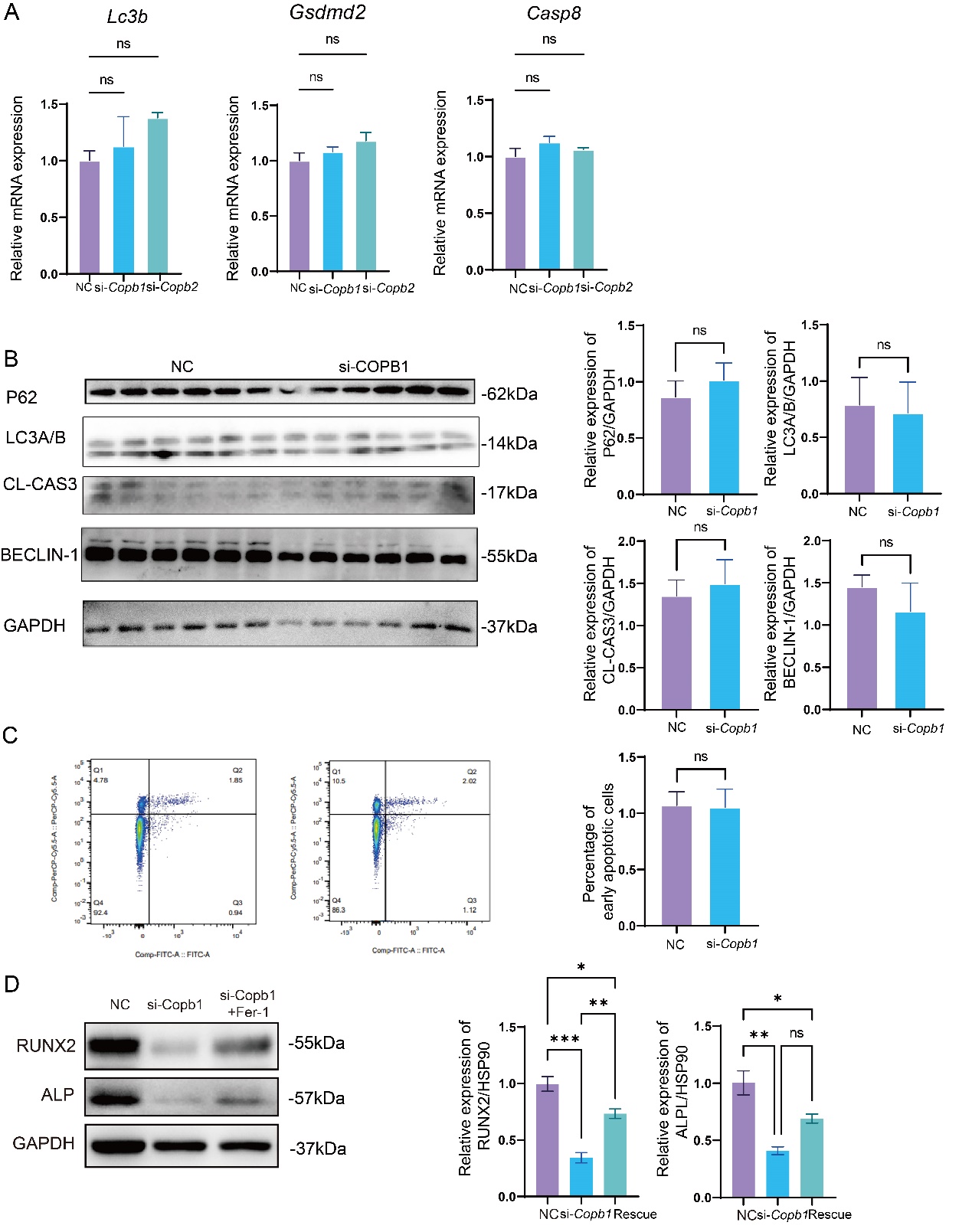


**Supplementary Figure 3**

a. qPCR analysis of autophagy (Lc3b), apoptosis (Caspase8), and pyroptosis (Gsdmd) in MC3T3-E1 cells in the control and knockdown groups after three days of knockdown (n=3 ). b. Western Blot analysis and quantitative statistical analysis of autophagy and apoptosis-related proteins (P62, LC3A/B, Cleaved-CASPASE3, BECLIN-1) in MC3T3-E1 cells in the control and knockdown groups after three days of knockdown (n=3). c. Flow cytometry detection and quantitative analysis of the percentage of early apoptotic cells in MC3T3-E1 cells (n=3). d. Western Blot analysis and quantitative statistical analysis of osteogenesis-related proteins (ALPL, RUNX2) in MC3T3-E1 cells in the control, knockdown, and rescue groups after seven days of osteogenic differentiation induction (n=3).


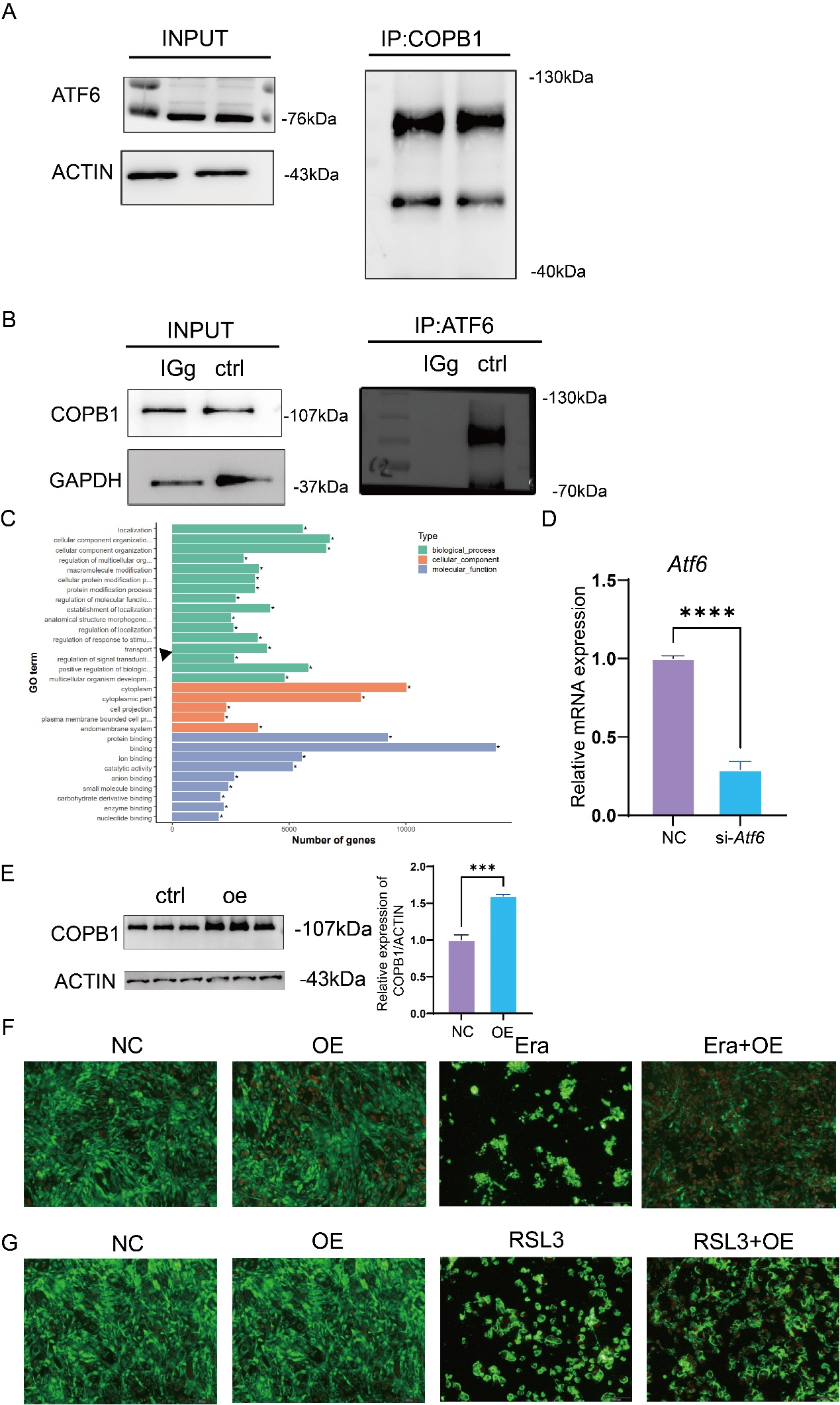


**Supplementary Figure 4**

a. CO-IP results of ATF6 using COPB1 antibody. b. CO-IP results of COPB1 using ATF6 antibody. c. CHIP-seq GO pathway enrichment analysis of ATF6. d. qPCR analysis of knockdown efficiency in MC3T3-E1 cells after three days of siRNA-mediated *Atf6* knockdown (n=3). e. Protein expression levels of COPB1 in MC3T3-E1 cells after lentiviral overexpression of COPB1 and quantitative statistical analysis(n=3). f. Immunofluorescence detection of live cell fluorescence intensity in MC3T3-E1 cells after overexpression of COPB1 and addition of Erastin. (n=3) g. Immunofluorescence detection of live cell fluorescence intensity in MC3T3-E1 cells after overexpression of COPB1 and addition of RSL3.(n=3)


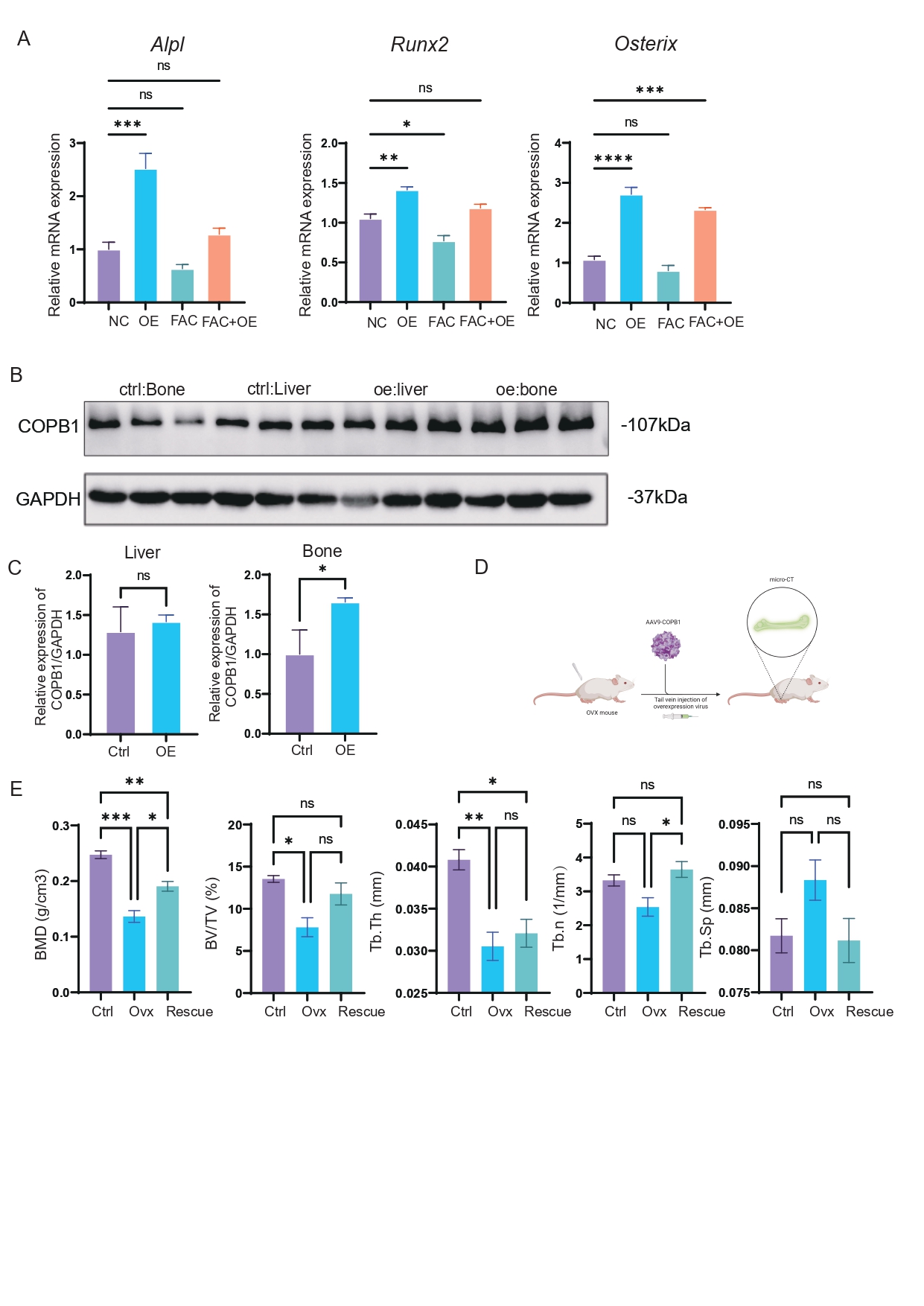


**Supplementary Figure 5**

a. qPCR analysis of ALPL, RUNX2, and OSX expression levels in the control, overexpression, iron accumulation, and rescue groups. (n=3) b, c. Western Blot analysis and quantitative statistical analysis of COPB1 expression levels in the liver and bone tissue of Ctrl and OE mice (n=3). d. Schematic diagram of the rescue experiment in Ovx mice. e. Histomorphometric analysis of trabecular bone in 4-month-old Ctrl, Ovx, and Rescue female mice, including bone mineral density (BMD), bone volume per tissue volume (BV/TV), trabecular thickness (Tb.Th), trabecular number (Tb.N), and trabecular separation (Tb.Sp) (n=3). )
